# Dietary Fiber Intake and Femoral Bone Mineral Density in Middle-Aged and Older US Adults: A Cross-Sectional Study of National Health and Nutrition Examination Survey 2013–2014

**DOI:** 10.3389/fnut.2022.851820

**Published:** 2022-03-14

**Authors:** Yuchen Tang, Jinmin Liu, Xiaohui Zhang, Bin Geng

**Affiliations:** ^1^Department of Orthopaedics, Lanzhou University Second Hospital, Lanzhou, China; ^2^Orthopaedics Key Laboratory of Gansu Province, Lanzhou, China; ^3^Orthopaedic Clinical Research Center of Gansu Province, Lanzhou, China

**Keywords:** dietary fiber, dietary fiber intake, bone mineral density, sex, femoral neck

## Abstract

Sufficient dietary fiber intake (DFI) is considered necessary for human health. However, the association between DFI and bone mineral density (BMD) remains unclear. Therefore, this study aimed to investigate the association between DFI and BMD and to determine whether sex modifies the association between DFI and BMD. Participants aged ≥ 40 years from the 2013–2014 National Health and Nutrition Examination Survey were included in the final analysis. The association between DFI and BMD was evaluated using a multivariate linear regression model. The non-linear relationship between DFI and BMD was characterized by smooth curve fittings and generalized additive models. Finally, 1,935 participants with a mean age of 58.12 ± 11.84 years were included in the final analysis. The results revealed that DFI was positively associated with femoral BMD in the unadjusted model. However, no correlation was observed between DFI and femoral BMD after adjusting for covariates. Moreover, the results showed an inverted U-shaped association between total DFI and femoral BMD among men but not women for the nonlinear relationship between DFI and femoral BMD. In conclusion, our results indicate that DFI might not follow a linear relationship with femoral BMD, and sex factors might modify the association between DFI and BMD. Particularly, high total DFI might contribute to lower femoral neck BMD. However, more studies are needed to investigate whether the negative effect of high DFI on femoral BMD does exist and whether high DFI has clear biological effects on bone metabolism, such as increasing the risk of osteoporosis.

## Introduction

Osteoporosis, characterized by reduced bone mineral density (BMD) and bone tissue microstructure degradation, is a common chronic disease worldwide (1). Approximately one-third of women and one-fifth of men aged ≥ 50 years are at risk of osteoporosis globally (1–3). Moreover, osteoporotic fracture, the most serious complication of osteoporosis, is also an important cause of death in older adults (4, 5). The pathogenesis of osteoporosis is complex and it is generally accepted that osteoporosis is determined by numerous genes and environmental factors (1). In addition, lifestyle factors play essential roles in the pathogenesis of osteoporosis (1, 6). For example, sufficient calcium or vitamin D intake is considered a key factor in the maintenance of bone mass (6, 7). Additional evidence has demonstrated that intake of other nutritional elements also essentially contribute to maintaining normal BMD, except for calcium and vitamin D. Therefore, exploring the impact of nutritional element intake on bone metabolism is receiving increasing attention, and it is expected to open novel avenues to prevent bone loss.

Dietary fiber (DF) is a carbohydrate polymer with ten or more monomeric units, which are not hydrolyzed by endogenous enzymes in the small intestine of humans and are typically derived from whole-grain cereals, fruits, vegetables, and legumes (8, 9). Several previous studies have shown that adequate DF intake (DFI) is necessary for disease prevention. Tanaka et al. observed that increased DFI reduces the incidence of stroke (10). Fujii et al. demonstrated that increased DFI is associated with better glycemic control and a lower risk of chronic kidney disease in patients with type 2 diabetes (11). Ananthakrishnan et al. found that adequate long-term DFI is associated with the decreased risk of Crohn's disease (12). Although the number is limited, related studies on bone metabolism have found that DFI might be associated with BMD (13–16). Dai et al. observed that increased DFI was associated with less bone loss among males but not females (14). Lee and Suh found that DFI was positively associated with lumbar BMD in men aged 18–45 years, but this correlation was not observed among women regardless of age (15). Zhou et al. demonstrated that higher DFI was associated with higher heel BMD among individuals aged 40–69 years, regardless of sex (16). Conversely, Barron et al. observed that a higher DFI was associated with lower lumbar BMD among young female athletes with oligomenorrhea (13). These contradicting findings suggest that the relationship between DFI and BMD remains unclear. Moreover, there was no definite evidence of whether sex modified the association between DFI and BMD.

Therefore, this study aimed to investigate the association between DFI and BMD. Moreover, we also tried to determine whether sex modified the association between DFI and BMD.

## Materials and Methods

### Study Population

We extracted data from the National Health and Nutrition Examination Survey (NHANES) database (2013–2014) (17). The NHANES database, affiliated with the Centers for Disease Control and Prevention (USA), aimed to assess the health and nutritional status of US residents and was updated biannually. Participants aged ≥ 40 years (the BMD test was only performed among participants aged ≥ 40 years in the NHANES 2013–2014) with complete data on BMD and DFI were enrolled in the present study. Moreover, subjects with missing covariate data (see details in the Covariates section) were excluded from the study. Each participant included in the present study obtained and signed the informed consent, and the Ethics Review Board of the National Center for Health Statistics approved the study (18).

### Bone Mineral Density Testing

All participants underwent BMD testing, which was based on the dual energy X-ray absorptiometry scan and assessed the BMD of four femoral regions (total femur, femoral neck, trochanter, and intertrochanter). Moreover, certified radiologic technologists conducted the dual-energy X-ray absorptiometry examinations using Hologic QDR-4500A fan-beam densitometers (Hologic; Bedford, MA), and the data analysis was performed using the Hologic APEX, version 4.0, software. Other details are available from the NHANES website (19).

### Dietary Fiber Intake

NHANES assessed the types and amounts of foods and beverages (including all types of water) consumed during the 24 h before the interview and estimated the DFI from those foods and beverages. In this study, the DFI referred to total DFI from the above foods and beverages. Information on DFI was collected through in-person interviews and telephone surveys (3–10 days after the in-person interview). The dietary recall statuses were classified as follows (i) reliable and met the minimum criteria; (ii) not reliable or did not meet the minimum criteria; (iii) reported consuming breast milk (for infants); and (iv) not done. In the present study, we enrolled only participants with a dietary recall status that was “reliable and met the minimum criteria” in the final analysis. Moreover, to balance the errors in both methods (in person or by phone), we calculated the mean values between the two and used them as the final values of DFI. Other details about the measurement of DFI are listed on the NHANES website (20, 21).

### Covariates

Considering that there were several factors that affected bone metabolism, we included covariates in the present study. Based on some previous studies (1, 22, 23), this study included the following covariates: age, sex, race, education level, income level, body mass index (BMI), smoking status, alcohol consumption, hypertension, diabetes, blood calcium level, serum 25-hydroxyvitamin D, rheumatoid arthritis (RA), cancer, use of glucocorticoid, family history of osteoporosis, previous fractures, physical activity level, calcium intake level, and vitamin D intake level. The specific information on the covariates is provided in Supplementary Table 1.

### Statistical Analysis

The baseline characteristics were described using the mean (for continuous variables) or proportion (for categorical variables). The linear relationship between DFI and BMD was assessed by multivariate linear regression models, while the non-linear relationship between DFI and BMD was evaluated by smooth curve fitting and generalized additive models. Moreover, if the non-linear relationship shows that an inflection point might exist, the inflection point can be calculated using two-piecewise linear regression models by a recursive algorithm. All analyses were performed using R software (version 4.0.3; https://www.R-project.org) and EmpowerStats (version 2.0; http://www.empowerstats.com). Statistical significance was set at *P* < 0.05.

## Results

### Participant Selection and Baseline Characteristics

**Figure 1:**
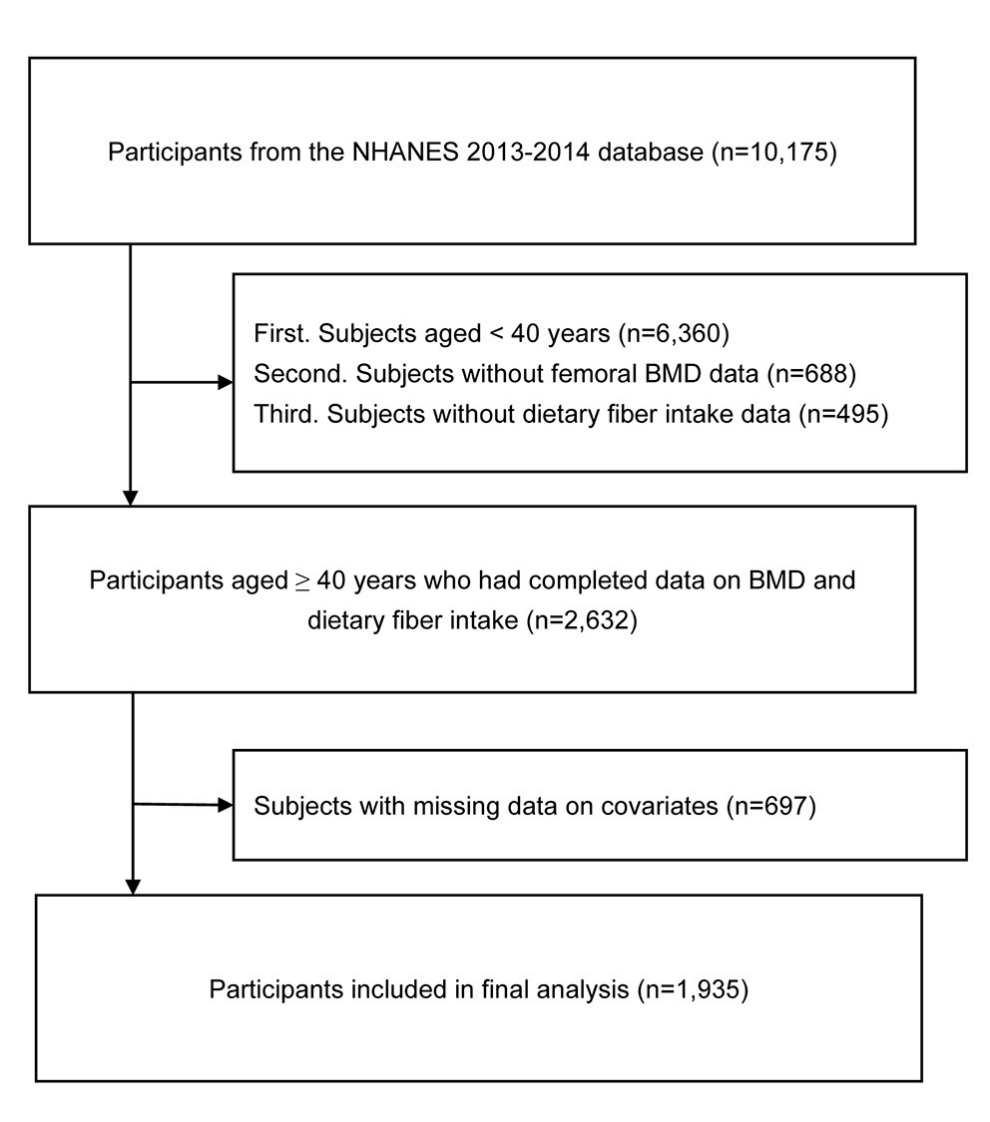
Flow chart of participant selection. NHANES, National Health and Nutrition Examination Survey; BMD, bone mineral density.

We extracted data from 10,175 participants from the NHANES (2013–2014) database. First, subjects aged <40 years (*n* = 6,360) were excluded from the present study. Second, subjects without femoral BMD data (*n* = 688) were also excluded. Third, subjects without dietary fiber intake data (*n* = 495) were excluded from this study. In addition, we excluded 697 subjects with missing data (missing data; refused to answer; or answered “do not know”) on covariates (Supplementary Figure 1). Finally, 1,935 participants were included in the final analysis. A flowchart of participant selection is shown in Figure 1.

The mean age of included participants was 58.12 ± 11.84 years. Moreover, most participants were females (50.96%), non-Hispanic whites (48.42%), had above high school education (59.07%), and were with ≥ 1 of poverty-income ratio (84.50%). In addition, the ratios of cases who were obese, current smoker, consumed at least 12 alcoholic drinks in the previous year, and were with diabetes, hypertension were 37.21, 16.90, 73.33, 44.81, 15.50%, respectively. Besides, the mean total femur BMD, femoral neck BMD, trochanter BMD, and intertrochanter BMD were 0.95 ± 0.15 g/cm^2^, 0.78 ± 0.14 g/cm^2^, 0.72 ± 0.12 g/cm^2^, 1.13 ± 0.18 g/cm^2^, respectively. Other details of the baseline characteristics are listed in Table 1.

**Table 1:** Baseline characteristics of included participants.

**Characteristics**		**Mean or proportion**
Age (year)		58.12 ± 11.84
Sex *n*, (%)	Male	949 (49.04%)
	Female	986 (50.96%)
Race *n*, (%)	Mexican American	249 (12.87%)
	Other hispanic	168 (8.68%)
	Non-hispanic white	937 (48.42%)
	Non-hispanic black	358 (18.50%)
	Other race	223 (11.52%)
Education level *n*, (%)	Under high school	371 (19.17%)
	High school or equivalent	421 (21.76%)
	Above high school	1,143 (59.07%)
Income level *n*, (%)	PIR <1	300 (15.50%)
	PIR ≥ 1	1,635 (84.50%)
BMI *n*, (%)	Normal	535 (27.65%)
	Overweight	680 (35.14%)
	Obesity	720 (37.21%)
Smoking status *n*, (%)	Current smokers	327 (16.90%)
	Quit smoking	563 (29.10%)
	Never	1,045 (54.01%)
Alcohol consumption *n*, (%)	Yes	1,419 (73.33%)
	No	516 (26.67%)
Hypertension *n*, (%)	Yes	867 (44.81%)
	No	1,068 (55.19%)
Diabetes *n*, (%)	Yes	300 (15.50%)
	No	1,560 (80.62%)
	Borderline	75 (3.88%)
Blood calcium level *n*, (%)	Q1: 8.2–9.1 (mg/dL)	382 (19.74%)
	Q2: 9.2–9.3 (mg/dL)	396 (20.47%)
	Q3: 9.4–9.6 (mg/dL)	655 (33.85%)
	Q4: 9.7–12.0 (mg/dL)	502 (25.94%)
Serum 25-hydroxyvitamin D *n*, (%)	Q1: 9.37–50.90 (nmol/L)	484 (25.01%)
	Q2: 51.00–67.20 (nmol/L)	476 (24.60%)
	Q3: 67.30–85.60 (nmol/L)	488 (25.22%)
	Q4: 85.70–318.00 (nmol/L)	487 (25.17%)
RA *n*, (%)	Yes	119 (6.15%)
	No	1,816 (93.85%)
Cancer *n*, (%)	Yes	252 (13.02%)
	No	1,683 (86.98%)
Use of glucocorticoid *n*, (%)	Yes	109 (5.63%)
	No	1,826 (94.37%)
Family history of osteoporosis *n*, (%)	Yes	286 (14.78%)
	No	1,649 (85.22%)
Previous fractures *n*, (%)	Yes	532 (27.49%)
	No	1,403 (72.51%)
Physical activity level *n*, (%)	NMVPA	486 (25.12%)
	LMVPA	295 (15.25%)
	MMVPA	232 (11.99%)
	HMVPA	922 (47.65%)
Calcium intake level *n*, (%)	Q1: 39.50–580.00 (mg/day)	484 (25.01%)
	Q2: 580.50–829.00 (mg/day)	483 (24.96%)
	Q3: 829.50–1,107.50 (mg/day)	484 (25.01%)
	Q4: 1,108.00–4,022.00 (mg/day)	484 (25.01%)
Vitamin D intake level *n*, (%)	Q1: 0.00–1.85 (mcg/day)	484 (25.01%)
	Q2: 1.90–3.50 (mcg/day)	475 (24.55%)
	Q3: 3.55–6.00 (mcg/day)	483 (24.96%)
	Q4: 6.05–46.30 (mcg/day)	493 (25.48%)
Total femur BMD (g/cm^2^)		0.95 ± 0.15
Femoral neck BMD (g/cm^2^)		0.78 ± 0.14
Trochanter BMD (g/cm^2^)		0.72 ± 0.12
Intertrochanter BMD (g/cm^2^)		1.13 ± 0.18

*BMI, body mass index; PIR, poverty-income ratio; RA, rheumatoid arthritis; HMVPA, high moderate-to-vigorous physical activity (≥1,200 MET-mins/week); MMVPA, medium moderate-to-vigorous physical activity (600–1,199 MET-mins/week); LMVPA, low moderate-to-vigorous physical activity (1–599 MET-mins/week); NMVPA, no moderate-to-vigorous physical activity (0 MET-mins/week)*.

### Association Between DFI and BMD

The results of multivariate linear regression models showed that DFI was positively associated with total femur (β: 0.0011; 95% CI: 0.0004–0.0019), trochanter (β: 0.0007; 95% CI: 0.0001–0.0013), and intertrochanter (β: 0.0013; 95% CI: 0.0005–0.0022) BMD in Model 1 (unadjusted model). However, no correlation was observed between DFI and femoral BMD after adjusting for covariates (Model 2 and Model 3). The specific results are shown in Table 2.

**Table 2:** Association between dietary fiber intake and femoral BMD.

**Index**	**Model 1**	**Model 2**	**Model 3**
	**β** **(95% CI)**	**β** **(95% CI)**	**β** **(95% CI)**
Total femur BMD	**0.0011 (0.0004, 0.0019)**	0.0002 (−0.0004, 0.0009)	0.0003 (−0.0003, 0.0010)
Femoral neck BMD	0.0006 (−0.0000, 0.0013)	0.0003 (−0.0003, 0.0009)	0.0002 (−0.0004, 0.0009)
Trochanter BMD	**0.0007 (0.0001, 0.0013)**	0.0001 (−0.0005, 0.0006)	0.0002 (−0.0004, 0.0007)
Intertrochanter BMD	**0.0013 (0.0005, 0.0022)**	0.0002 (-0.0006, 0.0010)	0.0003 (−0.0005, 0.0012)

*Bold variables indicate P-value < 0.05. Model 1: unadjusted model; Model 2: age, sex, and race were adjusted; Model 3: age, sex, race, education level, income level, BMI, smoking status, alcohol consumption, hypertension, diabetes, blood calcium level, serum 25-hydroxyvitamin D, RA, cancer, use of glucocorticoid, family history of osteoporosis, previous fractures, physical activity level, calcium intake level, and vitamin D intake level were adjusted. BMD, bone mineral density; CI, confidence interval; BMI, body mass index; RA, rheumatoid arthritis*.

When the variable of DFI was converted into a categorical variable, the results of multivariate linear regression models revealed that participants with the higher quartile of DFI (Q3 and Q4) had higher femoral BMD than those with the lowest quartile of DFI in Model 1 (unadjusted model). After adjusting for age, sex, and race (Model 2), the results revealed that participants with the third quartile of DFI showed higher total femur (β: 0.0187; 95% CI: 0.0023–0.0352) and trochanter (β: 0.0183; 95% CI: 0.0044–0.0323) BMD compared with those with the lowest quartile of DFI. When all covariates were adjusted (Model 3), participants with the third quartile of DFI still showed higher trochanter (β: 0.0147; 95% CI: 0.0013–0.0281) BMD than those with the lowest quartile of DFI, and no significant differences were observed in other groups. The specific results are listed in Table 3.

**Table 3:** Association between dietary fiber intake and femoral BMD.

**Index**	**Group**	**Model 1**	**Model 2**	**Model 3**
		**β** **(95% CI)**	**β** **(95% CI)**	**β** **(95% CI)**
Total femur BMD	Q1: 0.15–11.25 gm/day	Reference (0)	Reference (0)	Reference (0)
	Q2: 11.30–15.90 gm/day	0.0153 (−0.0038, 0.0343)	0.0106 (−0.0057, 0.0270)	0.0021 (−0.0129, 0.0172)
	Q3: 15.95–22.05 gm/day	**0.0199 (0.0009, 0.0389)**	**0.0187 (0.0023, 0.0352)**	0.0141 (−0.0016, 0.0297)
	Q4: 22.10–95.20 gm/day	**0.0340 (0.0150, 0.0530)**	0.0132 (−0.0037, 0.0300)	0.0131 (−0.0041, 0.0304)
Femoral neck BMD	Q1: 0.15–11.25 gm/day	Reference (0)	Reference (0)	Reference (0)
	Q2: 11.30–15.90 gm/day	0.0018 (−0.0161, 0.0197)	0.0022 (−0.0132, 0.0176)	−0.0058 (−0.0206, 0.0090)
	Q3: 15.95–22.05 gm/day	0.0059 (−0.0120, 0.0237)	0.0129 (−0.0026, 0.0284)	0.0072 (−0.0082, 0.0227)
	Q4: 22.10–95.20 gm/day	**0.0198 (0.0019, 0.0377)**	0.0131 (−0.0028, 0.0289)	0.0093 (−0.0077, 0.0263)
Trochanter BMD	Q1: 0.15–11.25 gm/day	Reference (0)	Reference (0)	Reference (0)
	Q2: 11.30–15.90 gm/day	0.0147 (−0.0007, 0.0302)	0.0117 (−0.0021, 0.0256)	0.0042 (−0.0087, 0.0170)
	Q3: 15.95–22.05 gm/day	**0.0189 (0.0035, 0.0343)**	**0.0183 (0.0044, 0.0323)**	**0.0147 (0.0013, 0.0281)**
	Q4: 22.10–95.20 gm/day	**0.0247 (0.0092, 0.0401)**	0.0111 (−0.0032, 0.0253)	0.0117 (−0.0030, 0.0265)
Intertrochanter BMD	Q1: 0.15–11.25 gm/day	Reference (0)	Reference (0)	Reference (0)
	Q2: 11.30–15.90 gm/day	0.0148 (−0.0079, 0.0375)	0.0084 (−0.0113, 0.0281)	−0.0006 (−0.0189, 0.0177)
	Q3: 15.95–22.05 gm/day	0.0200 (−0.0026, 0.0427)	0.0170 (−0.0029, 0.0368)	0.0114 (−0.0076, 0.0305)
	Q4: 22.10–95.20 gm/day	**0.0379 (0.0152, 0.0606)**	0.0110 (−0.0093, 0.0314)	0.0113 (−0.0097, 0.0323)

*Bold variables indicate P-value < 0.05. Model 1: unadjusted model; Model 2: age, sex, and race were adjusted; Model 3: age, sex, race, education level, income level, BMI, smoking status, alcohol consumption, hypertension, diabetes, blood calcium level, serum 25-hydroxyvitamin D, RA, cancer, use of glucocorticoid, family history of osteoporosis, previous fractures, physical activity level, calcium intake level, and vitamin D intake level were adjusted. BMD, bone mineral density; CI, confidence interval*.

### Association Between DFI and BMD Stratified by Sex

**Figure 2:**
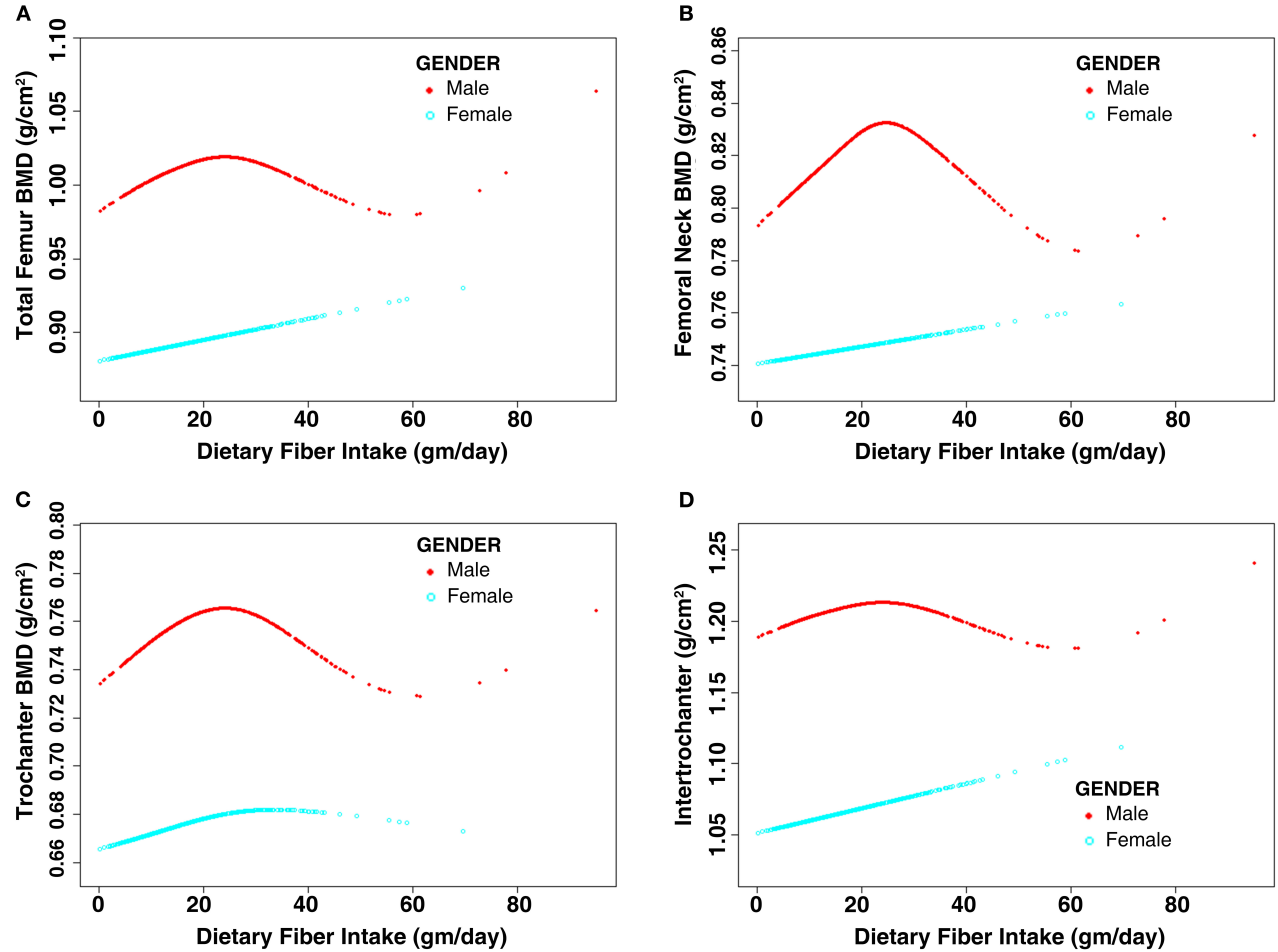
Non-linear relationship between dietary fiber intake and femoral BMD stratified by sex. Age, race, education level, income level, BMI, smoking status, alcohol consumption, hypertension, diabetes, blood calcium level, serum 25-hydroxyvitamin D, RA, cancer, use of glucocorticoid, family history of osteoporosis, previous fractures, physical activity level, calcium intake level, and vitamin D intake level were adjusted. **(A)** Total femur BMD; **(B)** Femoral neck BMD; **(C)** Trochanter BMD; **(D)** Intertrochanter BMD. BMD, bone mineral density; BMI, body mass index; RA, rheumatoid arthritis.

The subgroup analysis stratified by sex is shown in Table 4. The results of multivariate linear regression models revealed that DFI was not associated with femoral BMD (*P* > 0.05) regardless of sex. Moreover, further analysis of the non-linear relationship between DFI and femoral BMD showed an inverted U-shaped association between DFI and femoral BMD among men but not women, and the inflection points of DFI observed were about 25 gm/day (Figure 2). In addition, the two-piecewise linear regression models demonstrated the inverted *U*-shaped association between DFI and femoral BMD among men. In particular, DFI was negatively associated with femoral neck BMD (β: −0.0017; 95% CI: −0.0032 to −0.0002) among men when DFI was >25 gm/day. The details are listed in Table 5.

**Table 4:** Association between dietary fiber intake and femoral BMD stratified by sex.

**Sex**	**Index**	**Model 1**	**Model 2**	**Model 3**
		**β** **(95% CI)**	**β** **(95% CI)**	**β** **(95% CI)**
Male	Total femur BMD	0.0000 (−0.0009, 0.0009)	0.0002 (−0.0006, 0.0011)	−0.0001 (−0.0010, 0.0008)
	Femoral neck BMD	0.0000 (−0.0009, 0.0009)	0.0003 (−0.0005, 0.0012)	−0.0002 (−0.0011, 0.0008)
	Trochanter BMD	−0.0001 (−0.0008, 0.0006)	0.0001 (−0.0006, 0.0008)	−0.0000 (−0.0008, 0.0008)
	Intertrochanter BMD	−0.0001 (−0.0011, 0.0009)	0.0002 (−0.0009, 0.0012)	−0.0002 (−0.0013, 0.0009)
Female	Total femur BMD	0.0000 (−0.0010, 0.0011)	0.0002 (−0.0007, 0.0012)	0.0008 (−0.0002, 0.0018)
	Femoral neck BMD	−0.0002 (−0.0012, 0.0008)	0.0002 (−0.0007, 0.0011)	0.0005 (−0.0005, 0.0015)
	Trochanter BMD	−0.0002 (−0.0010, 0.0007)	0.0000 (−0.0008, 0.0008)	0.0004 (−0.0004, 0.0012)
	Intertrochanter BMD	0.0001 (−0.0012, 0.0014)	0.0003 (−0.0009, 0.0015)	0.0010 (−0.0002, 0.0022)

*Model 1: unadjusted model; Model 2: age and race were adjusted; Model 3: age, race, education level, income level, BMI, smoking status, alcohol consumption, hypertension, diabetes, blood calcium level, serum 25-hydroxyvitamin D, RA, cancer, use of glucocorticoid, family history of osteoporosis, previous fractures, physical activity level, calcium intake level, and vitamin D intake level were adjusted. BMD, bone mineral density; CI, confidence interval; BMI, body mass index; RA, rheumatoid arthritis*.

**Table 5:** Two-piecewise linear regression models of dietary fiber intake on BMD in males.

**Index**	**Total femur BMD**	**Femoral neck BMD**	**Trochanter BMD**	**Intertrochanter BMD**
Fitting by the standard linear model	−0.0001 (−0.0010, 0.0008)	−0.0002 (−0.0011, 0.0008)	−0.0000 (−0.0008, 0.0008)	−0.0002 (−0.0013, 0.0009)
Fitting by the two-piecewise linear model				
Inflection point (gm/day)	25	25	25	25
Dietary fiber intake < Infection point	0.0011 (−0.0005, 0.0027)	0.0015 (−0.0001, 0.0031)	0.0012 (−0.0002, 0.0025)	0.0007 (−0.0012, 0.0026)
Dietary fiber intake > Infection point	−0.0011 (−0.0026, 0.0004)	−0.0017 (−0.0032, −0.0002)	−0.0012 (−0.0025, 0.0002)	−0.0010 (−0.0028, 0.0008)

*Age, race, education level, income level, BMI, smoking status, alcohol consumption, hypertension, diabetes, blood calcium level, serum 25-hydroxyvitamin D, RA, cancer, use of glucocorticoid, family history of osteoporosis, previous fractures, physical activity level, calcium intake level, and vitamin D intake level were adjusted. BMD, bone mineral density; BMI, body mass index; RA, rheumatoid arthritis*.

## Discussion

Osteoporosis in middle-aged and older individuals has become a global issue in the past decade. Currently, there is an increasing awareness that dietary changes or lifestyle modifications might be an effective mean of preventing osteoporosis. This study found that DFI was positively associated with femoral BMD in the unadjusted model. However, no correlation was observed between DFI and femoral BMD after adjusting for covariates. For the non-linear relationship between DFI and femoral BMD, the results showed an inverted *U*-shaped association between DFI and femoral BMD among males but not females. In addition, DFI was negatively associated with femoral neck BMD among males when DFI was >25 gm/day.

This study found that DFI was positively associated with femoral BMD in the unadjusted model, but no correlation was observed between DFI and femoral BMD after adjusting for covariates. This finding seemed to be the important differences compared with the existing literature (14–16). Dai et al. observed that increased DFI was associated with reduced bone loss in men (14). Moreover, Lee and Suh found that DFI was positively associated with lumbar BMD in men aged 18–45 years (15). Zhou et al. demonstrated that a higher DFI was associated with higher heel BMD among individuals aged 40–69 years (16). There are several possible explanations for the discrepancy between our study and previous study. First, DFI may not be associated with BMD. DF is derived from whole-grain cereals, fruits, vegetables, and legumes, and these foods also contain other nutritional elements such as calcium and vitamin D, which are considered to play essential roles in maintaining bone mass (6, 7). High DFI might also be associated with high calcium or vitamin D intake, which might be a potential reason for the discrepancy between the present and previous studies. Therefore, we initially included the variables of calcium and vitamin D intake as covariates to avoid potential bias. However, our study found that no correlation was observed between DFI and femoral BMD after adjusting for all covariates. Second, DFI might be associated with BMD, but our study did not observe this because of the limitations of the present study. On the one hand, the DFI data were collected based on short-term intake, using short-term dietary intake as usual intake, to assess the association between DFI and femoral BMD; this might lead to a biased estimate of the association. Therefore, these findings also suggest that further studies on the relationship between DFI and BMD need to consider the influence of exposure time. Meanwhile, the information on DFI was collected based on self-report in the present study, which is a subjective parameter and might not reflect the actual DFI. Third, DFI might be associated with BMD, but the relationship between DFI and BMD was influenced by other factors, such as age, sex, or anatomical sites. In the present study, the association between DFI and BMD seemed to be modified by sex. Similarly, the association between DFI and BMD may be modified by other factors. For example, Lee and Suh found that DFI was associated with BMD in men aged 18–45 years but not in those aged over 65 years. Dai et al. observed that total DFI was correlated with femoral neck BMD but not lumbar BMD (14). Considering the limited number of related studies, additional studies are needed to confirm our hypothesis.

This study also observed sex differences in the association between DFI and femoral BMD. We considered that the sex differences might have resulted from hormone levels, especially sex hormones. Barron et al. observed that higher DFI was associated with lower lumbar BMD among young female athletes with oligomenorrhea (13), which is a symptom possibly caused by disorders of sex hormones. These findings combined with the results of our study suggested that DF might play various roles in different sex hormone levels. In addition, the impact of DF on the gut microbiota may have sex differences. Zhang et al. observed sex differences in the gut microbiome in response to DF supplementation in experimental animals (24). Similarly, Morrison et al. found a sex-specific effect of DFI on the gut microbiota community composition in animal experiments (25). However, there is no direct evidence supporting our hypotheses, and the mechanisms remain ambiguous. Therefore, further studies are needed to investigate this intriguing observation.

Interestingly, this study observed that DFI was negatively associated with femoral neck BMD among men when DFI was >25 gm/day, suggesting that high DFI might be unfavorable to prevent bone loss or even contribute to lower femoral BMD. We considered that there were some underlying mechanisms of high DFI leading to low BMDs. First, a high DFI might contribute to low femoral BMDs by altering the composition of the intestinal microbiota. Actually, high DFI could indeed alter the composition of the intestinal microbiota (26, 27). Moreover, cumulative evidence indicates that the gut microbiota is linked to bone metabolism (28, 29). However, further studies on the impact of DF on bone metabolism are needed to support our hypotheses because direct proof has been missing. Second, high DFI might contribute to low femoral BMDs by affecting hormone levels, such as estrogen levels. Wayne et al. found that high DFI was associated with low serum estradiol levels among postmenopausal breast cancer survivors (30). Similarly, Zengul et al. observed an inverse association between DFI and estradiol levels in postmenopausal women with breast cancer (31). However, these studies did not prove that DFI could directly affect estrogen metabolism, and no evidence has demonstrated that the inverse association between DFI and estrogen levels exists among middle-aged and older men. Therefore, whether high DFI might contribute to low femoral BMDs by reducing estrogen levels is an interesting topic for further study. Third, high DFI might contribute to low femoral BMDs by enhancing intestinal inflammation and affecting calcium and vitamin D absorption. Grabitske and Slavin suggested that a higher or excessive fiber intake might cause gastrointestinal effects, such as diarrhea and abdominal discomfort (32). Miles et al. demonstrated that the addition of inulin, a DF, exacerbated the severity of colitis induced by dextran sulfate sodium in mice (33). However, these studies did not directly prove our hypotheses, and the number of related studies is limited. Moreover, there were also several studies demonstrated that high DFI might be a protective factor for inflammatory bowel disease (34, 35). In addition, it remains unclear whether the negative correlation between DFI and femoral BMD has clear biological effects, such as increasing the risk of osteoporosis. Therefore, additional research is needed to explore whether high DFI contributes to lower femoral BMD or whether the negative effect of high DFI on femoral BMD only applies to specific populations.

The findings of the present study could also provide references or guidelines for daily routine practice and future research. Specifically, the findings of this study might provide a reference for the recommended intake of DF, especially in high-risk population. According to the 2020–2025 Dietary Guidelines for Americans (36), individuals aged 31–50 years and those aged over 50 years should consume at least 31 and 28 g of DF per day, respectively. In the present study, we observed that DFI was negatively associated with femoral neck BMD among men when DFI was >25 gm/day. Therefore, to prevent bone loss, excess DFI might not be appropriate for middle-aged and older men. However, high DFI might also be a protective factor against other diseases, such as coronary artery disease, cancer, and diabetes (37, 38). The number of studies on the impact of DF on bone metabolism was limited. Therefore, additional prospective studies are needed to determine the optimal threshold of DF intake. On the other hand, the findings of the present study might also provide a reference for future research on the relationship between DFI and bone metabolism. Except for the negative association between DFI and femoral BMD among men with high DFI (>25 gm/day), this study also observed sex differences in the relationship of DFI with femoral BMD between men and women. Although more studies are needed to investigate whether the negative correlation between DFI and femoral BMD has clear biological effects, such as increasing the risk of osteoporosis, this study offers a new perspective on the potential impact of DF on bone metabolism.

This study had some limitations. First, the DFI data were collected based on short-term intake, using short-term dietary intake as usual intake to assess the association between DFI and femoral BMD, which might lead to a biased estimate of the association. Second, the final analysis was based on individuals with complete data. Subjects with missing data were excluded from the present study, which might have produced bias. Third, the DFI data were collected based on subjective self-reports. Therefore, there might be some discrepancy between self-reported DFI and actual DFI. Fourth, the participants included in the final analysis were based on the general US population. Considering the differences in culture, lifestyle, and diet among different countries and regions, more studies are needed to investigate whether the conclusions of the present study are generally applicable. Finally, some unmeasured confounding variables (such as bone turnover markers), which are also considered important factors for bone metabolism, were not assessed in the present study because these variables were not available in the NHANES database, and the lack of adjustment for these potential factors may have biased the results.

In conclusion, our results indicate that DFI might not follow a linear relationship with femoral BMD, and sex factors might modify the association between DFI and BMD. In particular, high DFI (>25 gm/day) might contribute to lower femoral neck BMDs among males aged ≥ 40 years. However, more studies are needed to investigate whether the negative effect of high DFI on femoral BMD does exist and whether high DFI has clear biological effects on bone metabolism, such as increasing the risk of osteoporosis.

## Data Availability Statement

The original contributions presented in the study are included in the article/Supplementary Material, further inquiries can be directed to the corresponding author/s.

## Ethics Statement

The studies involving human participants were reviewed and approved by NCHS Research Ethics Review Board. The patients/participants provided their written informed consent to participate in this study.

## Author Contributions

YT and JL conceived the study, data curation, data analysis, and draft writing. YT and XZ completed images and tables preparation. BG conceived the study, funding acquisition, and writin—review and editing. All authors participated in critical revision of the manuscript, contributed to the article, and approved the submitted version.

## Funding

This work was supported by the National Natural Science Foundation of China (81960403), Cuiying Scientific and Technological Innovation Program of Lanzhou University Second Hospital (CY2021-MS-A07), and Innovation Star Project for Excellent Graduate Students of the Education Department of Gansu Province (2021CXZX-143).

## Conflict of Interest

The authors declare that the research was conducted in the absence of any commercial or financial relationships that could be construed as a potential conflict of interest.

## Publisher's Note

All claims expressed in this article are solely those of the authors and do not necessarily represent those of their affiliated organizations, or those of the publisher, the editors and the reviewers. Any product that may be evaluated in this article, or claim that may be made by its manufacturer, is not guaranteed or endorsed by the publisher.
