# Supplementary material for: Dietary Fiber Intake and Femoral Bone Mineral Density in Middle-Aged and Older US Adults: A Cross-Sectional Study of National Health and Nutrition Examination Survey 2013–2014
Source: Front Nutr. 2022 Mar 14;9:851820. doi: 10.3389/fnut.2022.851820 (PMC8964086; doi:10.3389/fnut.2022.851820)
Supplement: Supplementary file 1 [file Data_Sheet_1.docx]

Supplementary Material


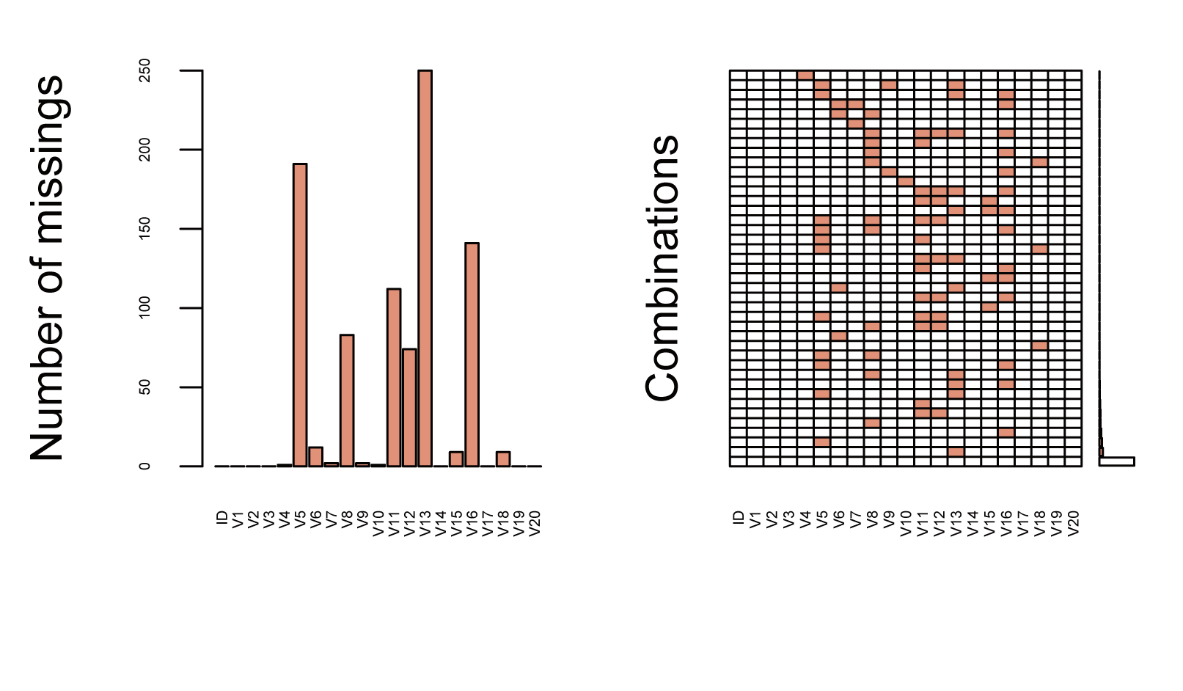


**Supplementary Figure 1.** Missing data on covariates. V1, age; V2, sex; V3, race; V4, education level; V5, income to poverty ratio; V6, BMI; V7, smoking status; V8, alcohol consumption status; V9, hypertension; V10, diabetes; V11, blood calcium; V12, serum 25-hydroxyvitamin D; V13, RA; V14, cancer or malignancy; V15, glucocorticoid use; V16, family history of osteoporosis; V17, history of fractures. V18, physical activity level; V19, vitamin D intake; V20, calcium intake. BMI, body, mass index; RA, rheumatoid arthritis.

**Supplementary Table 1** Detailed information on covariates

| **Covariate** | **Range (if continues variable)** | **Grouping** |
| --- | --- | --- |
| **Age** | 40-80 years ("80" means ≥ 80 years of age) | 40-49 years |
|  |  | 50-59 years |
|  |  | 60-69 years |
|  |  | 70-79 years |
|  |  | ≥ 80 years |
| **Sex** | NA | Male |
|  |  | Female |
| **Race** | NA | Mexican American |
|  |  | Other Hispanic |
|  |  | Non-Hispanic White |
|  |  | Non-Hispanic Black |
|  |  | Other Race - Including Multi-Racial |
| **Education level** | NA | Under high school |
|  |  | High school or equivalent |
|  |  | Above high school |
| **Income level (poverty-income ratio)** | 0.00-5.00 ("5.00" means ≥ 5.00) | PIR < 1 |
|  |  | PIR ≥ 1 |
| **BMI** | 14.20-65.50 kg/m2 | Normal (BMI < 25kg/m2) |
|  |  | Overweight (25 ≤ BMI < 30kg/m2) |
|  |  | Obesity (BMI ≥ 30kg/m2) |
| **Smoking status** | NA | Current smokers |
|  |  | Quit smoking |
|  |  | Never |
| **Alcohol consumption** | NA | Yes (had at least 12 alcohol drinks past year) |
|  |  | No |
| **Hypertension** | NA | Yes (diagnosed by doctors) |
|  |  | No |
| **Diabetes** | NA | Yes (diagnosed by doctors) |
|  |  | No |
|  |  | Borderline |
| **Blood calcium level** | 8.2-12.0 mg/dL | Q1: 8.2-9.1 (mg/dL) |
|  |  | Q2: 9.2-9.3 (mg/dL) |
|  |  | Q3: 9.4-9.6 (mg/dL) |
|  |  | Q4: 9.7-12.0 (mg/dL) |
| **Serum 25-hydroxyvitamin D** | 9.37-318.00 nmol/L | Q1: 9.37-50.90 (nmol/L) |
|  |  | Q2: 51.00-67.20 (nmol/L) |
|  |  | Q3: 67.30-85.60 (nmol/L) |
|  |  | Q4: 85.70-318.00 (nmol/L) |
| **RA** | NA | Yes (Diagnosed with RA by doctors) |
|  |  | No |
| **Cancer** | NA | Yes (Diagnosed with cancer by doctors) |
|  |  | No |
| **Use of glucocorticoid** | NA | Yes (Had glucocorticoid use) |
|  |  | No |
| **Family history of osteoporosis** | NA | Yes (patients with osteoporosis diagnosed by doctors) |
|  |  | No |
| **Previous fractures** | NA | Yes (self-report) |
|  |  | No |
| **Physical activity level** | 0-42240 MET-mins/week | HMVPA (≥ 1200 MET-mins/week) |
|  |  | MMVPA (600–1199 MET-mins/week) |
|  |  | LMVPA (1–599 MET-mins/week) |
|  |  | NMVPA (0 MET-mins/week) |
| **Calcium intake** | 39.50-4022.00 mg/day | Q1: 39.50-580.00 (mg/day) |
|  |  | Q2: 580.50-829.00 (mg/day) |
|  |  | Q3: 829.50-1107.50 (mg/day) |
|  |  | Q4: 1108.00-4022.00 (mg/day) |
| **Vitamin D intake** | 0.00-46.30 mcg/day | Q1: 0.00-1.85 (mcg/day) |
|  |  | Q2: 1.90-3.50 (mcg/day) |
|  |  | Q3: 3.55-6.00 (mcg/day) |
|  |  | Q4: 6.05-46.30 (mcg/day) |

NA, not applicable; BMI, body mass index; PIR, poverty-income ratio; RA, rheumatoid arthritis.
